# A Dual-Marker DNA Methylation Assay Enables High-Sensitivity Detection of Malignant Effusions Across Cancer Types

**DOI:** 10.3390/cancers18193176

**Published:** 2026-10-01

**Authors:** Ting Wang, Ting Liang, Matthew Kafarski, Jian Liu, Yi Jing, Wen Liao, Hang Xing, Jia Feng, Tingting Bian, Lei Liu, Hui Sun, Xiaoli Zheng, Bin She, Xiaosha Ren, Joanne Lee, Qiushi Lin, Ming Chen, Liang Cheng, Hong Ge, Xiaoqun Dong, Yifei Liu

**Affiliations:** 1 Department of Radiation Oncology, The Affiliated Cancer Hospital of Zhengzhou University & Henan Cancer Hospital, No. 127 Dongming Road, Jinshui District, Zhengzhou 450008, China; wangting199007@126.com (T.W.); ziye0705@163.com (X.Z.); zlyygehong0199@zzu.edu.cn (H.G.); 2 Department of Pathology, Affiliated Hospital of Nantong University, Medical School of Nantong University, Nantong 226001, China; 2331310138@stmail.ntu.edu.cn (T.L.); jingyii0618@163.com (Y.J.); liaowen0026@163.com (W.L.); tdfyjessicavon@ntu.edu.cn (J.F.); biantingting@ntu.edu.cn (T.B.); liulei19920123@163.com (L.L.); sunhui0509@163.com (H.S.); 3 Precision Health Program, Department of Radiology, College of Human Medicine, Michigan State University, East Lansing, MI 48824, USA; kafarsk1@msu.edu (M.K.); linqius1@msu.edu (Q.L.); chenmi26@msu.edu (M.C.); 4 Department of Oncology, Affiliated Hospital of Nantong University, Medical School of Nantong University, No. 20 Xisi Road, Chongchuan District, Nantong 226001, China; niuniu68915@sina.com; 5 Rhode Island Hospital, Alpert Medical School of Brown University, Providence, RI 02912, USA; hang_xing@brown.edu; 6 Director of Pathology Business Unit, Shanghai ZJ Bio-Tech CO., Ltd., Building 9, No. 2168 Chenhang Hwy, Shanghai 201114, China; shebin_cn@163.com (B.S.); xiaosha4769@163.com (X.R.); 7 Department of Pathology and Laboratory Medicine, Brown University Warren Alpert Medical School, 142 Boren Street, Providence, RI 02912, USA; joanne_lee1@brown.edu (J.L.); liang_cheng@brown.edu (L.C.)

**Keywords:** DNA methylation, *SHOX2*, *RASSF1A*, malignant effusion, epigenetics

## Abstract

**Objective:** This study evaluated the diagnostic value of a combined *SHOX2* and *RASSF1A* methylation assay (LungMe^®^) in differentiating malignant from benign pleural and peritoneal effusions. **Methods:** A total of 696 patients with pleural or peritoneal effusions were prospectively enrolled. Effusion sediment samples were analyzed by liquid-based cytology (TCT), cell block histology, and methylation-specific PCR for both *SHOX2* and *RASSF1A*. The final diagnosis was confirmed by clinical, imaging, and pathological follow-up. **Results:** Lung cancer was the predominant cause of malignant pleural effusions (71.96%), while malignant peritoneal effusions mainly resulted from ovarian, gastric, and liver cancers. The optimal ΔCt cutoffs for *SHOX2* and *RASSF1A* were ΔCt ≤ 9 and ΔCt ≤ 12, respectively. The combined methylation assay achieved a sensitivity of 79.1% and specificity of 90.4% for malignant effusions. When combined with cytology, the overall sensitivity increased from 34.7% (TCT alone) to 91.7%, with a positive predictive value (PPV) of 93.9% and a negative predictive value (NPV) of 86.7%. The assay showed high sensitivity across multiple tumor types, including 100% for esophageal and breast cancers, and ≥80% for lung cancer, gastric cancer, lymphoma, and cholangiocarcinoma. **Conclusions:** The combined SHOX2 and RASSF1A methylation assay has high diagnostic value for benign–malignant discrimination of pleural and peritoneal effusions, improving diagnostic sensitivity when combined with conventional cytology. Thus, within the context of this single-center study, the LungMe^®^ assay demonstrates promise in expanding pan-cancer diagnostics, but will require further multicenter external validation and prospective clinical studies before routine clinical adoption.

## Simple Summary

Distinguishing malignant from benign pleural and peritoneal effusions is essential for cancer diagnosis and treatment, but routine cytology often fails to detect malignant cells. We evaluated a DNA methylation test based on two cancer-related genes, *SHOX2* and *RASSF1A*, in 696 patients with pleural or peritoneal effusions. The test showed higher sensitivity than conventional cytology while maintaining high specificity, and combining the two methods further improved diagnostic accuracy across multiple cancer types. The assay was particularly useful for identifying cancers that were missed or considered uncertain by cytological examination. These findings suggest that dual-gene DNA methylation testing is a reliable and minimally invasive tool that can complement routine pathology, improve the diagnosis of malignant effusions, and support earlier clinical decision-making for patients with cancer.

## 1. Introduction

Pleural and peritoneal effusions (ascites), or the abnormal accumulation of fluid in the pleural and abdominal cavities, arise from a wide range of pathological conditions, including malignancies, infections, heart failure, tuberculosis, and cirrhosis [1,2,3]. Accurate differentiation between benign and malignant effusions is critical, as it directly influences clinical management, treatment decisions, and prognosis, yet current diagnostic approaches remain unable to reliably make this distinction [4,5].

Cytological examinations and thoracoscopic pleural biopsy remain the gold standards for diagnosis for pleural and peritoneal effusions [6]. However, the sensitivity of conventional cytology is limited (typically 40–60%), particularly in samples with low cellularity or equivocal morphology, largely due to reactive mesothelial cells that can mimic malignancy [7,8]. While liquid-based cytology (TCT) and cell block preparations have improved sample quality and detection rates [9], significant diagnostic gaps remain, especially for ascites [10]. Thus, a molecular diagnostic test that combines high sensitivity with robust specificity is urgently needed to reliably distinguish malignant from benign effusions across cancer types and support clinical evaluation.

DNA methylation is a key epigenetic mechanism in tumorigenesis and has emerged as a promising cancer biomarker [11,12]. The short stature homeobox 2 (*SHOX2*) gene is frequently hypermethylated in various cancers, including lung, breast, and gastrointestinal tumors [13,14]. Additionally, the Ras association domain family protein 1A (*RASSF1A*) is a tumor suppressor gene whose promoter hypermethylation is an established independent predictor of tumor invasion, metastasis, and poor prognosis in lung, gastrointestinal, breast, and head & neck cancers [15,16,17].

The LungMe^®^ assay, which detects combined *SHOX2* and *RASSF1A* methylation, has demonstrated particularly high diagnostic accuracy for lung cancer using bronchoalveolar lavage fluid (sensitivity 71.5–84.0%, specificity 80.7–90.0%) [18,19]. In pleural effusions, this dual-marker approach achieved even higher performance for detecting malignant effusions (sensitivity 71.4–96%, specificity 84.8–100%) [20,21]. However, its clinical utility has not been systematically evaluated across both pleural and peritoneal effusions from a broad spectrum of malignancies. Addressing this gap, the present study prospectively investigates the diagnostic performance of this dual-marker methylation signature (alone and in combination with cytomorphology) for differentiating benign from malignant effusions in a large, diverse patient cohort.

## 2. Materials and Methods

### 2.1. The Study Design and Patient Selection

From June 2021 to October 2023, a total of 696 consecutive, de-identified patients with pleural or peritoneal effusions were prospectively enrolled at The Affiliated Hospital of Nantong University. The final diagnosis of effusion (benign vs. malignant) was established by a composite reference standard based on clinical presentation, imaging, cytological/histological examination of fluid and/or biopsy tissue, and follow-up data (minimum 6 months for benign diagnoses). Patients with uncertain final diagnoses were excluded. The study protocol was approved by the Institutional Review Board of The Affiliated Hospital of Nantong University and was conducted in accordance with the Declaration of Helsinki.

### 2.2. Sample Collection and Processing

For each patient, 50–200 mL of fresh pleural or ascitic fluid was collected. A 5–10 mL aliquot was centrifuged at 2000 rpm for 10 min. The cell pellet was resuspended in ThinPrep cytology test (TCT) preservation solution for cytologic examination. The remaining fluid was similarly centrifuged, and the cell pellet was processed into a formalin-fixed, paraffin-embedded (FFPE) cell block for histopathologic evaluation, as previously described [9].

### 2.3. Cytology (TCT) and Cell Block Histology

TCT slides were prepared using a ThinPrep 2000 processor (Hologic, Marlborough, MA, USA), stained with Papanicolaou or hematoxylin–eosin (H&E), and examined by experienced cytopathologists. FFPE cell blocks were sectioned (4 μm), stained with H&E, and examined by experienced histopathologists. All cytological and histological interpretations were performed as part of routine clinical care, with pathologists blinded to the methylation results.

### 2.4. DNA Extraction and Methylation-Specific PCR (MS-PCR)

Genomic DNA was extracted from the cell pellet (from the remaining fluid after TCT aliquot) using the Methy-All-In-One Kit (Shanghai Tellgen Life Science & Technology Co., Ltd., Shanghai, China). DNA concentration was measured fluorometrically. Bisulfite conversion of 200 ng DNA was performed using the Methy-All-In-One Kit (Shanghai Tellgen Life Science & Technology Co., Ltd., Shanghai, China). The converted DNA was eluted in 20 μL, and 5 μL was used as a template for MS-PCR.

The MS-PCR assay (LungMe^®^ kit, Shanghai Tellgen) simultaneously detects methylated *SHOX2* (VIC channel), methylated *RASSF1A* (FAM channel), and a reference gene, β-actin (CY5 channel). Real-time PCR was performed on an SLAN-96S system (Hongshi, Shanghai, China) using the thermal cycling conditions specified within the manufacturer’s instructions. Methylation levels were expressed as ΔCt = Ct(target gene) − Ct(β-actin), with a lower ΔCt indicating a higher methylation level.

To ensure assay reproducibility, quality control and analytical validation were performed using the following criteria and methods. A sample was considered valid if the β-actin Ct value was <28 (optimal range 18–23), with samples exceeding this threshold being subject to retesting. Positive and negative controls (provided with the kit) were included in each run, together with three control samples representing high, medium, and low methylation levels, each tested in duplicate. Inter-run variability, calculated as the coefficient of variation (CV) for ΔCt values across 20 independent runs, was <5% for all three control levels. All 696 clinical samples met the β-actin validity criteria, signifying a 0.00% assay failure rate, and no repeat testing of invalid results was required.

### 2.5. Statistical Analysis

The primary outcome measures were sensitivity, specificity, positive predictive value (PPV), and negative predictive value (NPV), each reported with 95% confidence intervals (CIs) calculated using the Clopper–Pearson (exact) method. ROC curves were generated using the ΔCt values of *SHOX2* and *RASSF1A* as test variables and the final clinical diagnosis as the state variable. The optimal cutoff was determined by maximizing the Youden index. A logistic regression model was built to combine the raw ΔCt values of both markers, and the predicted probability from the model was used to generate a combined ROC curve. The AUCs were compared using the DeLong test. All statistical analyses were performed using SPSS version 26.0 (IBM Corp., Armonk, NY, USA) and R software (R version 4.4.1, Statistical Computing, Vienna, Austria). A two-sided *p*-value < 0.05 was considered statistically significant.

### 2.6. Data Availability Statement

The datasets generated and analyzed during the current study are available from the corresponding author upon reasonable request.

## 3. Results

### 3.1. Patient Characteristics

A total of 696 patients with pleural or peritoneal effusions were enrolled in this study, including 476 cases of pleural effusion and 220 cases of ascites. Among these, 435 cases were malignant and 261 were benign. There were 407 males and 289 females. The proportion of male patients was slightly higher than that of females in both malignant and benign groups (Table 1).

The mean age of onset for benign and malignant effusions ranged from 64.1 to 68.8 years, with no statistically significant difference among groups. Analysis of age distribution showed that patients under 50 years of age had a significantly higher proportion of benign effusions compared to malignant effusions (19.9% vs. 6.0%, *p* < 0.05). The peak incidence of malignant effusions occurred in the 70–79 years age group (31.7%), while benign effusions were most common in the 60–69 years age group (23.0%) (Table 1).

**Figure 1:**
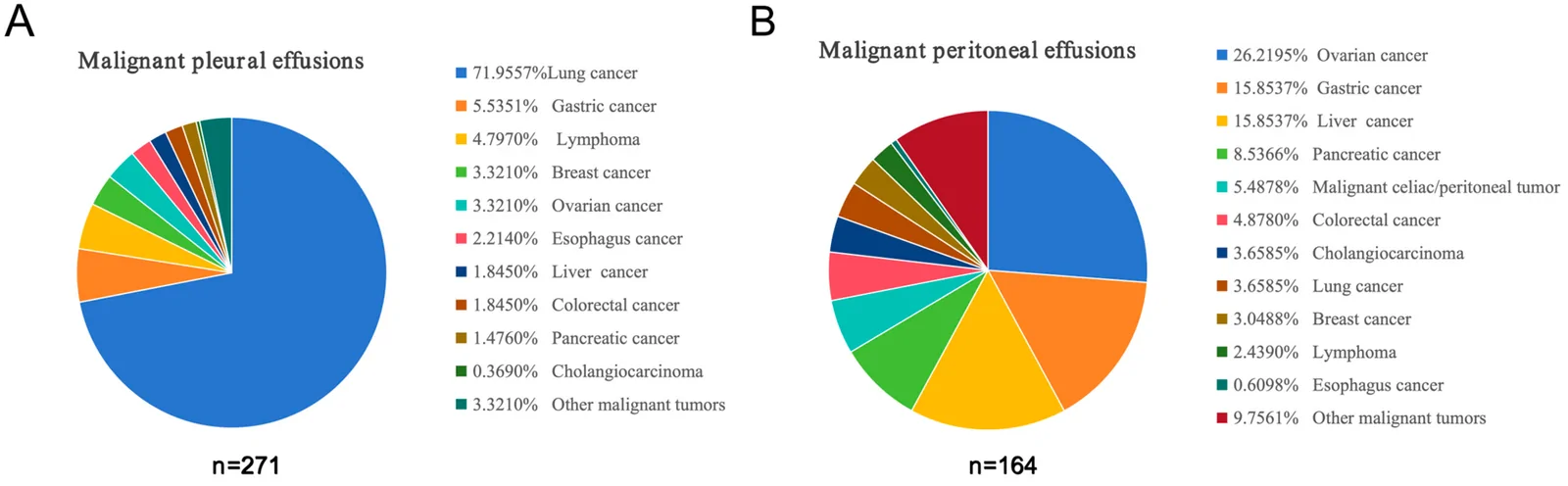
Distribution of tumor types causing malignant pleural effusions and peritoneal effusions. (**A**) Tumor types associated with malignant pleural effusions; (**B**) tumor types associated with malignant peritoneal effusions.

Lung cancer was the predominant cause of malignant pleural effusions (71.96%), followed by gastric cancer (5.54%), lymphoma (4.80%), and breast cancer (3.32%) (Figure 1A). The most frequent malignancies associated with malignant ascites were ovarian (26.22%), gastric (15.85%), liver (15.85%), and pancreatic cancer (8.54%) (Figure 1B).

### 3.2. Determination of Optimal ΔCt Cutoffs via ROC Analysis

Methylation-specific qPCR was performed for all samples, and ΔCt values (Ct_gene − Ct_β-actin) were calculated. Receiver operating characteristic (ROC) analysis was used to determine optimal cutoffs for distinguishing malignant from benign effusions, using the final clinical diagnosis as the reference standard. Youden’s index was applied to identify the optimal cutoff points.

**Figure 2:**
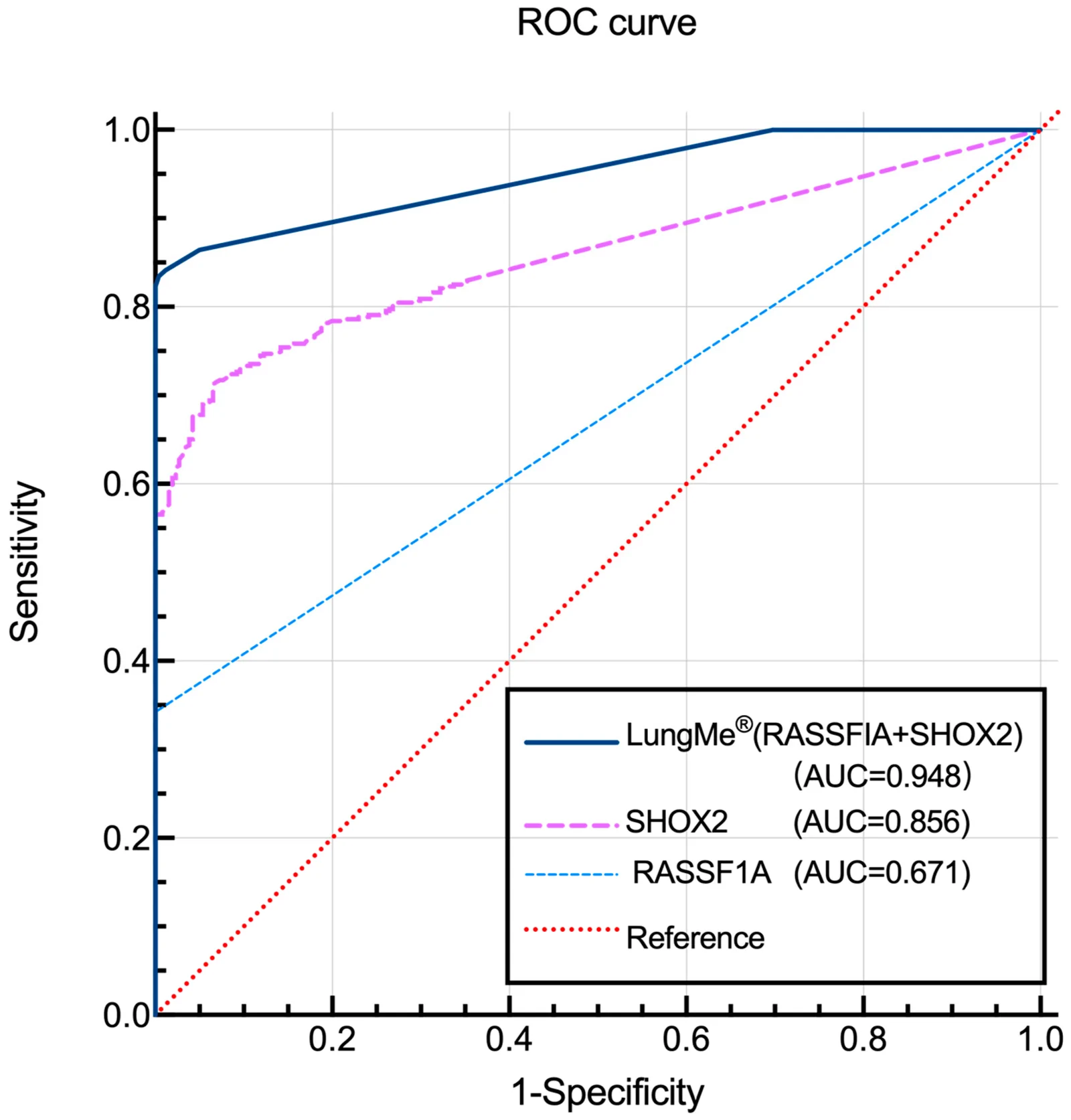
Diagnostic performance of *SHOX2* and *RASSF1A* methylation in pleural and peritoneal effusions assessed by ROC curve analysis.

For *SHOX2*, the optimal ΔCt cutoffs were 8.88 for pleural fluid and 8.46 for ascites. For simplicity and standardization, a unified cutoff of 9 was selected for both fluid types, which was justified as a cutoff located on the flat region of the ROC curve (e.g., 9.0) provided better generalizability. For *RASSF1A*, the optimal cutoffs were 17.33 (pleural) and 16.54 (ascites). However, the LungMe^®^ assay reliably detects *RASSF1A* methylation only when Ct < 35; beyond this threshold, reproducibility is compromised [22,23]. Since β-actin Ct values in clinical samples typically range from 18 to 23, a ΔCt cutoff of 12 corresponded to an *RASSF1A* Ct of 30–35 (within stable range) while a ΔCt cutoff of 16–17 would correspond to a Ct of 34–40, which was beyond the reliable limit of the LungMe^®^ assay. Therefore, ΔCt ≤ 12 was selected as a conservative cutoff for *RASSF1A* to ensure analytical robustness and maintain high specificity. The area under the ROC curve (AUC) for *SHOX2* alone was 0.856 (95% CI: 0.827–0.885), and for *RASSF1A* alone was 0.671 (95% CI: 0.631–0.711). A logistic regression model combining the raw ΔCt values of both markers achieved an AUC of 0.948 (95% CI: 0.930–0.966; *p* < 0.001 for comparison with *SHOX2* or *RASSF1A* alone), indicating the strong additive discriminatory potential of the dual-marker model (Figure 2). However, this exploratory continuous model differs from the predefined binary LungMe^®^ algorithm in which *SHOX2* ΔCt ≤ 9 or *RASSF1A* ΔCt ≤ 12 is sufficient for detection.

**Figure 3:**
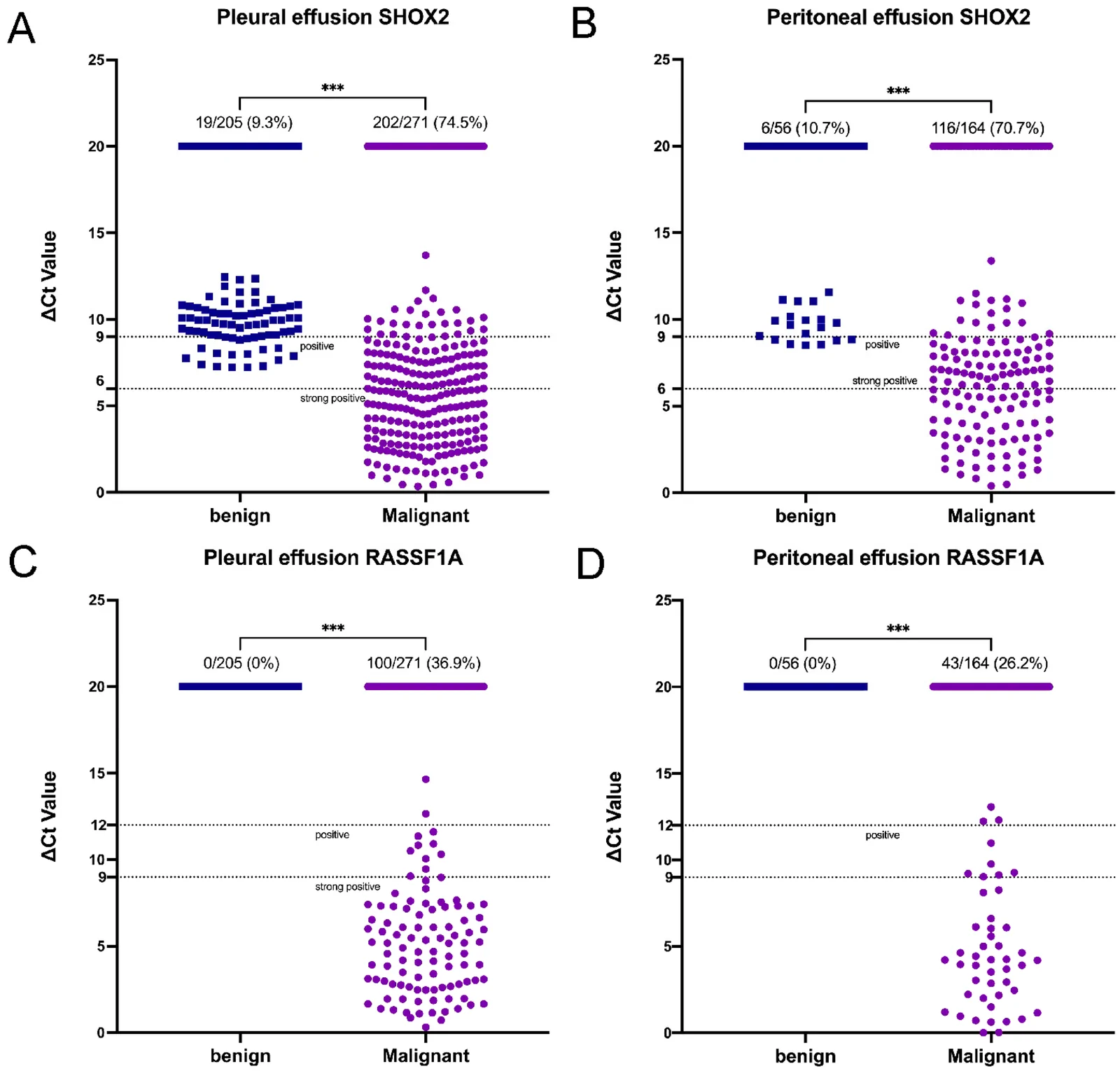
Scatter plots showing ΔCt distributions of *SHOX2* and *RASSF1A* methylation in pleural and peritoneal effusions. (**A**) ΔCt distribution of *SHOX2* in benign versus malignant pleural effusions. (**B**) ΔCt distribution of *SHOX2* in benign versus malignant peritoneal effusions. (**C**) ΔCt distribution of *RASSF1A* in benign versus malignant pleural effusions. (**D**) ΔCt distribution of *RASSF1A* in benign versus malignant peritoneal effusions. A ΔCt value of 20 indicates absence of methylation signal. Statistical significance: ***, *p* < 0.001.

Applying the unified cutoffs (*SHOX2* ΔCt ≤ 9; *RASSF1A* ΔCt ≤ 12), the sensitivity of *SHOX2* methylation was 74.5% (95% CI: 68.9–79.5%) for pleural and 70.7% (95% CI: 63.2–77.3%) for ascites, with specificities of 90.7% (95% CI: 85.9–94.0%) and 89.3% (95% CI: 78.1–95.0%), respectively (Figure 3A,B). In contrast, *RASSF1A* methylation showed lower sensitivity (pleural: 36.9%; ascites: 26.2%) but achieved markedly higher specificity (100%) (Figure 3C,D). Remarkably, when redefining *SHOX2* strong positivity as ΔCt ≤ 6 and *RASSF1A* strong positivity as ΔCt ≤ 9, a specificity for malignancy of 100% was achieved. These methylation thresholds (ΔCt ≤ 6 and ΔCt ≤ 9) were precisely 3 cycles lower than the initial cutoffs (ΔCt ≤ 9 and ΔCt ≤ 12, respectively), corresponding to an 8-fold increase in methylation levels and producing a substantial increase in diagnostic reliability.

### 3.3. Diagnostic Performance of Methylation vs. Cytology

The diagnostic performance of the LungMe^®^ methylation assay (positive if *SHOX2* ΔCt ≤ 9 OR *RASSF1A* ΔCt ≤ 12) was compared with TCT and cell block histology, using the final clinical diagnosis as the gold standard.

**Figure 4:**
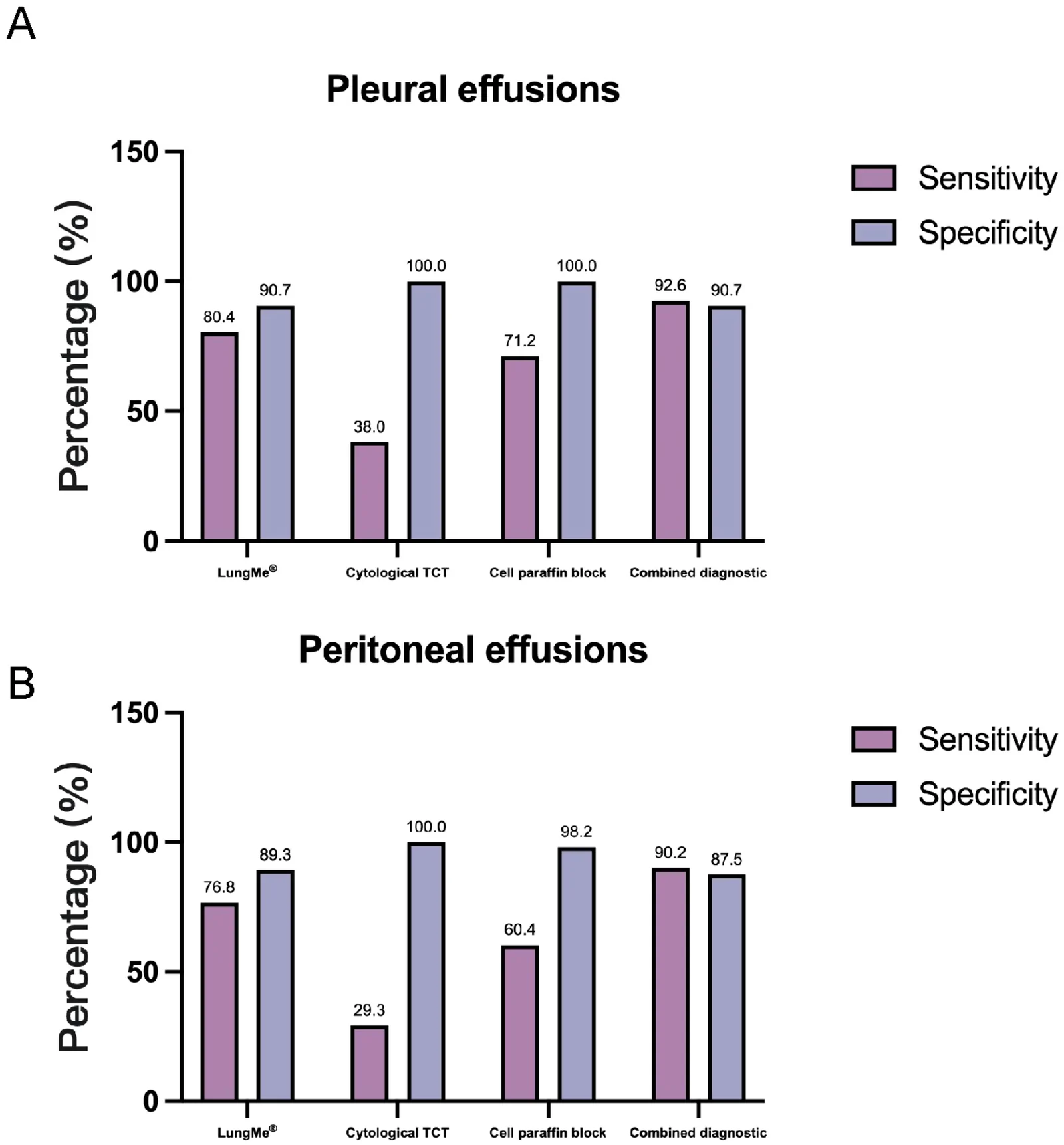
Sensitivity and specificity of molecular methylation versus cytology in distinguishing benign and malignant pleural and peritoneal effusions. (**A**) Comparison of sensitivity and specificity for methylation and cytology in diagnosing benign and malignant pleural effusions. (**B**) Comparison of sensitivity and specificity for methylation and cytology in diagnosing benign and malignant peritoneal effusions.

In pleural effusions (Figure 4A), the LungMe^®^ assay demonstrated a sensitivity of 80.4% (95% CI: 75.1–84.9%), significantly outperforming TCT (38.0%, 95% CI: 32.3–44.0%) and cell block histology (71.2%, 95% CI: 65.4–76.4%). In ascites (Figure 4B), methylation sensitivity was 76.8% (95% CI: 69.6–82.8%), compared to 29.3% (95% CI: 22.8–36.6%) for TCT and 60.4% (95% CI: 52.6–67.7%) for cell block.

Across all effusions (Table 2), the LungMe^®^ assay (sensitivity 79.1% [95% CI: 75.0–82.9%]) was superior to both TCT (34.7% [95% CI: 30.3–39.3%]) and cell block (67.1% [95% CI: 62.5–71.5%]). Specificity was high for all methods (TCT: 100%, cell block: 99.6%, LungMe^®^: 90.4% [95% CI: 86.2–93.6%]). The combination of LungMe^®^ with cytology (TCT or cell block, positive if any test positive) increased overall sensitivity to 91.7% (95% CI: 88.7–94.1%) while maintaining a specificity of 90.0% (95% CI: 85.7–93.4%). Importantly, a triple-negative result yielded an NPV of 86.7% (95% CI: 81.9–90.5%), substantially higher than TCT (47.9%), cell block (64.5%), or LungMe^®^ alone (72.2%). Nevertheless, approximately 13% of patients with all three tests negative may still have a malignant effusion, so clinical follow-up remains mandatory.

### 3.4. Resolving Equivocal and False-Negative Cytology Results

Cytology often yields “gray zone” results (e.g., atypical cells). In 273 cases (39.2%) with equivocal cytology, the positive predictive value (PPV) was only 68.5% (95% CI: 62.2–74.2%). Adding a positive LungMe^®^ result in these cases significantly improved the PPV to 89.6% (95% CI: 84.0–93.5%) and specificity to 93.5% (95% CI: 87.8–96.9%) (Table 2).

Furthermore, among the 435 patients with malignant effusions, TCT results were classified into three mutually exclusive categories: positive (*n* = 151), equivocal (*n* = 187), and negative (*n* = 97). Among the 97 malignant cases with negative cytology, LungMe^®^ yielded positive results in 74 cases and negative results in 23 cases (Supplementary Table S1). Among the 187 malignant cases with equivocal cytology, LungMe^®^ yielded positive results in 147 cases and negative results in 40 cases (Supplementary Table S2). Thus, malignant cases that were non-positive by TCT and negative by LungMe^®^ comprised 63 patients in total (40 with equivocal cytology and 23 with negative cytology). Of these, 22 of the 40 equivocal/LungMe^®^-negative cases and 14 of the 23 negative/LungMe^®^-negative cases were also negative by cell-block cytology, yielding a total of 36 malignant cases negative by all three methods. Accordingly, 399 of 435 malignant cases were detected by at least one of the three methods, corresponding to a combined sensitivity of 91.7%.

Among the 272 patients with negative cytology, 97 were classified as malignant and 175 as benign according to the reference standard. LungMe^®^ yielded positive results in 74 of the 97 malignant cases and in 8 of the 175 benign cases, whereas negative results were observed in the remaining 23 malignant cases and 167 benign cases. Thus, a total of 82 patients in the cytology-negative subgroup were LungMe^®^-positive, including 74 malignant and 8 benign cases (Supplementary Table S1). In this subgroup, LungMe^®^ demonstrated a sensitivity of 76.3% (74/97), a specificity of 95.4% (167/175), a positive predictive value (PPV) of 90.2% (74/82), and a negative predictive value (NPV) of 87.9% (167/190).

### 3.5. Diagnostic Value Across Different Tumor Types

Performance of the combined LungMe^®^ assay: The sensitivity of the LungMe^®^ assay for detecting malignant effusions varied across cancer types but remained high for most (Table 3). When analyzing pleural effusions and peritoneal effusions separately, the assay achieved 100% sensitivity for esophageal and breast cancers in both fluid types. Moreover, sensitivity exceeded 80% for lung, gastric, lymphoma, and cholangiocarcinoma while remaining moderate for ovarian (77.8% and 72.1%) and hepatocellular (60.0% and 73.1%) carcinomas across both pleural and peritoneal effusions. Interestingly, pancreatic cancer demonstrated the most pronounced fluid-type disparity (50.0% in pleural effusions and 85.7% in peritoneal effusions), though the imbalance in subgroup sizes (*n* = 4 and *n* = 14, respectively) limited interpretation. Lastly, the lowest sensitivity was observed for colorectal cancer (60.0% in pleural effusions, 50.0% in peritoneal effusions; overall 53.8%, 95% CI: 26.6–79.4%), though the sample size was small (*n* = 13). The combined assay demonstrated robust diagnostic performance across a broad spectrum of malignancies, supporting its clinical utility for pan-cancer effusion detection.

**Figure 5:**
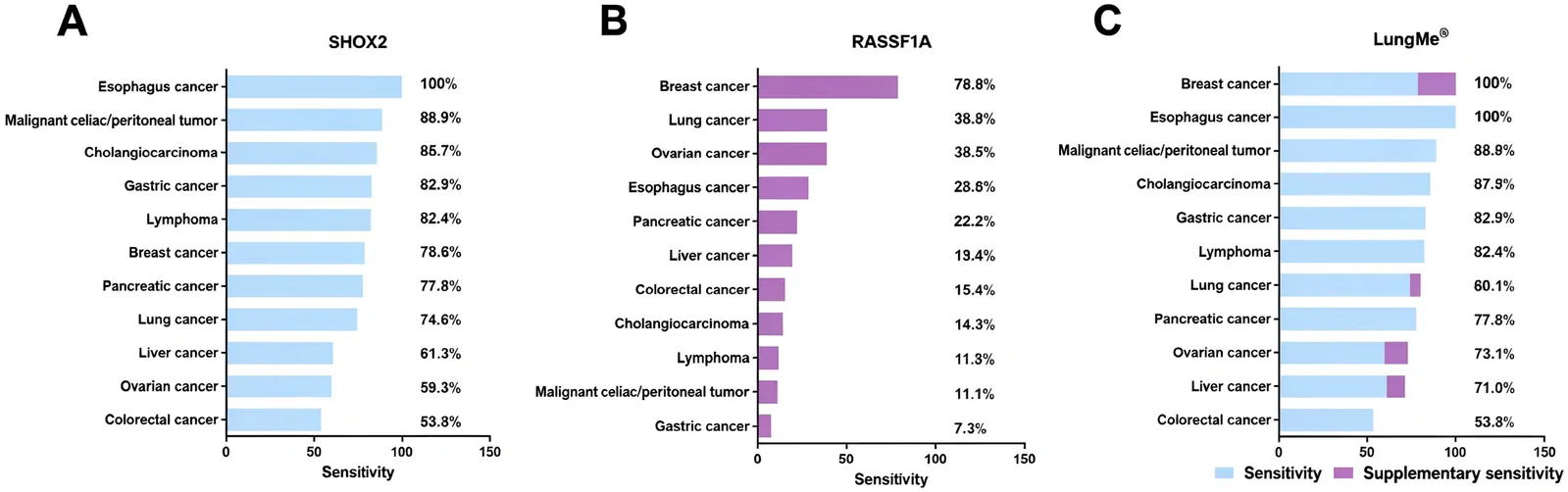
Diagnostic sensitivity of *SHOX2* and *RASSF1A*, individually and combined, in malignant pleural and peritoneal effusions across various tumor types. (**A**) Diagnostic sensitivity of *SHOX2* for malignant effusions caused by different tumors. (**B**) Diagnostic sensitivity of *RASSF1A* for malignant effusions caused by different tumors. (**C**) Diagnostic sensitivity of LungMe^®^ methylation assay (combined *SHOX2* and *RASSF1A*) for malignant effusions caused by different tumors.

Individual performance of *SHOX2* and *RASSF1A* methylation was also analyzed separately. *SHOX2* methylation alone exhibited high sensitivity for most tumor types, with the highest sensitivities observed for esophageal cancer, cholangiocarcinoma, gastric cancer, lymphoma, breast cancer, pancreatic cancer, and lung cancer, ranging from 74.6% to 100% (Figure 5A). Sensitivity was modestly lower for liver carcinoma (61.3%), ovarian carcinoma (59.3%), and colorectal carcinoma (53.8%) (Figure 5A). In contrast, *RASSF1A* methylation showed substantially lower overall sensitivity, with a positivity rate between 11.1% and 28.6% across most tumor types (Figure 5B). Notably, *RASSF1A* achieved a sensitivity of 78.6% in breast cancer, whereas it was only 38.8% in lung cancer, 38.5% in ovarian cancer, and as low as 7.3% in gastric cancer (Figure 5B). When the two markers were combined (LungMe^®^ assay), *RASSF1A* provided a complementary effect to SHOX2, particularly in breast cancer, ovarian cancer, liver cancer, and lung cancer (Figure 5C).

To further quantify the incremental contribution of *RASSF1A*, marker status was cross-tabulated across all 435 malignant cases, of which 203 (46.7%) were *SHOX2*-positive/*RASSF1A*-negative, 28 (6.4%) were *SHOX2*-negative/*RASSF1A*-positive, 115 (26.4%) were positive for both, and 89 (20.5%) were negative for both. *RASSF1A* therefore identified 28 malignant effusions missed by *SHOX2* alone, an absolute sensitivity gain of 6.4% (95% CI: 4.3–9.2%; McNemar’s test, *p* < 0.001). This gain was unevenly distributed across tumor types, being the greatest in breast cancer (2/14, +14.3%), followed by ovarian (4/52, +7.7%), liver (2/31, +6.5%), and lung (11/201, +5.5%) cancers, minimal in gastric cancer (1/41, +2.4%), and absent in colorectal cancer (0/13). Yet, the incremental *RASSF1A* yield was comparable between effusion types (pleural 18/271, +6.6%; peritoneal 10/164, +6.1%), suggesting that the added value of RASSF1A may be determined primarily by tumor lineage rather than by the serosal compartment sampled (Figure 5C).

## 4. Discussion

This large, prospective study systematically compared the diagnostic performance of a combined *SHOX2* and *RASSF1A* methylation assay (LungMe^®^) with conventional cytology (TCT) and cell block histology for differentiating benign from malignant pleural and peritoneal effusions across multiple cancer types. Our findings confirm the well-recognized limitation of cytology: high specificity but low sensitivity. In this cohort, TCT achieved a sensitivity of only 34.7% for all effusions (29.3% for peritoneal effusions and 38.0% for pleural fluid) at a specificity of 100%, which is consistent with previous reports [7,8]. Cell block examination significantly improved sensitivity to 67.1% overall (60.4% for peritoneal effusions and 71.2% for pleural fluid) at a specificity of 99.6%, but a considerable diagnostic gap remained, particularly for malignant peritoneal effusions. The LungMe^®^ methylation assay demonstrated markedly higher sensitivity (79.1% overall; 80.4% for pleural effusions and 76.8% for peritoneal effusions) while maintaining a specificity of 90.4%. Paired McNemar’s test confirmed that methylation testing detected significantly more malignant effusions than either TCT or cell block alone (*p* < 0.001 for both). However, the LungMe^®^ methylation assay also produced significantly more false-positive results among benign effusions (*p* < 0.001 for both), exchanging a modest loss of specificity for a substantial gain in sensitivity. These outcomes suggest that the use of a combined SHOX2 and RASSF1A methylation assay alone may be most effective as a first-line adjunct in the initial evaluation of effusions of undetermined etiology, where a false negative could result in delayed diagnosis and additional invasive testing. Yet, when LungMe^®^ methylation testing is combined with cytology (TCT and cell block), overall sensitivity increases to 91.7% without compromising specificity (90.0%), offering a powerful new diagnostic strategy worthy of further validation.

Our ROC analysis identified optimal ΔCt cutoffs of 9 for *SHOX2* and 12 for *RASSF1A*, which were consistent with findings from multiple previously studied independent cohorts [24,25]. This reproducibility across different laboratories and patient populations supports the potential standardization of this assay for clinical use. The observed sensitivity of the LungMe^®^ assay (80.4% for pleural effusions) aligns well with the 76.5% positive detection rate reported by Liang et al. [24], but is lower than the 96% sensitivity reported by Zhang et al. using cell-free DNA from pleural effusion supernatant [21]. This discrepancy highlights the ongoing debate regarding the optimal DNA source for methylation analysis, which we address in further detail.

**Figure 6:**
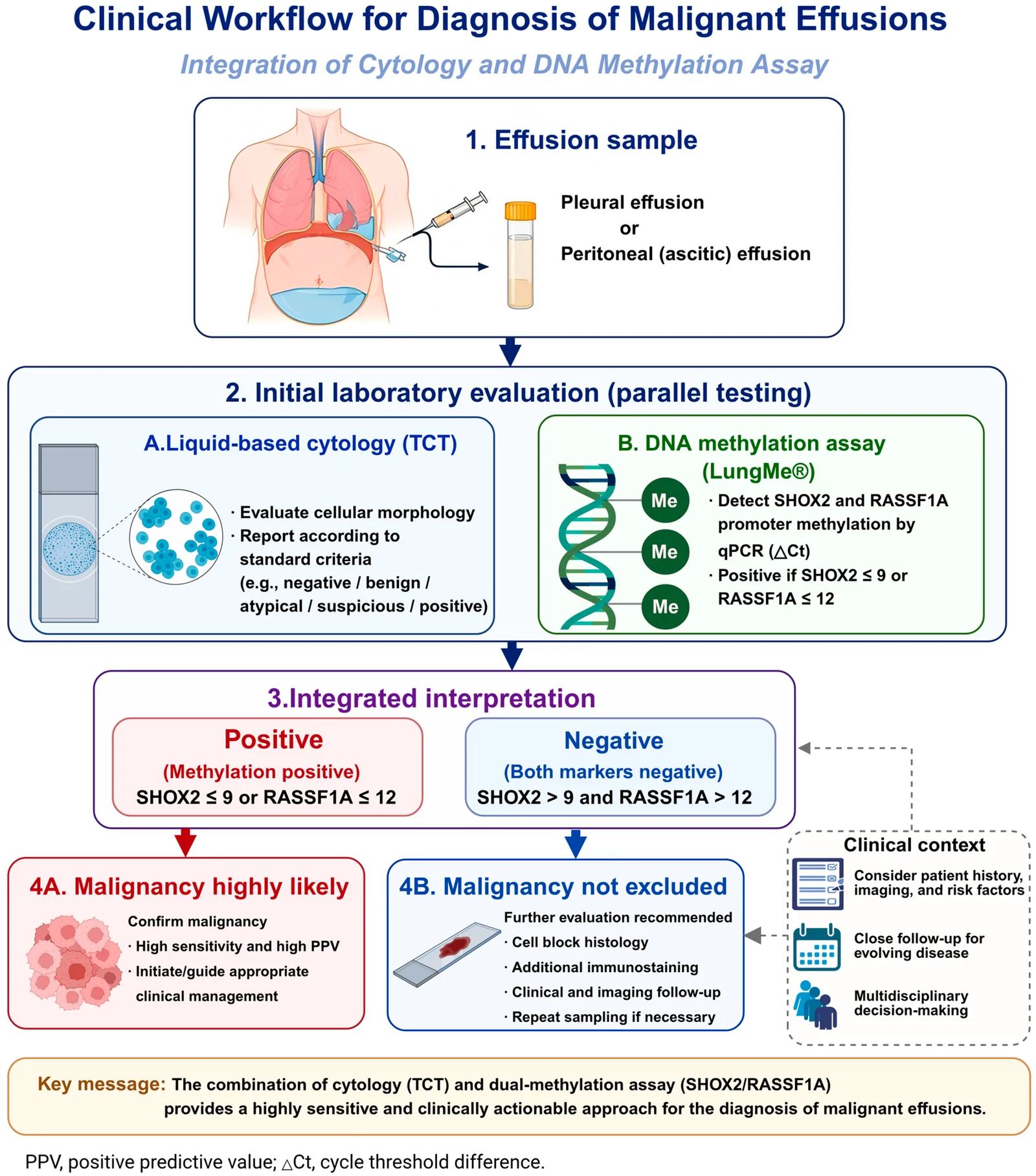
Integrated diagnostic workflow combining cytology and dual-gene methylation testing for malignant effusions.

To facilitate clinical translation of these findings, we propose a practical diagnostic workflow integrating cytology and methylation testing (Figure 6). First, fresh cell samples can undergo simultaneous TCT and methylation testing with a rapid turnaround time (typically within two working days). Positive or equivocal results can then trigger reflex testing, such as cell block preparation, H&E staining, and immunohistochemistry, for definitive diagnosis. Second, while morphological diagnosis offers near-perfect specificity, its limited sensitivity results in a high positive predictive value (PPV) alongside a low negative predictive value (NPV). Incorporating methylation testing addresses this gap: when all three tests (TCT, cell block, methylation) are negative, the NPV reaches 86.7%, providing strong reassurance to rule out malignancy. Third, for the substantial proportion of patients receiving an equivocal (“gray zone”) cytology report (39.2% in our study), a positive methylation result effectively resolves diagnostic ambiguity. In our cohort, adding methylation to equivocal cytology increased the PPV from 68.5% to 89.6% and specificity from 67.0% to 93.5%, enabling high-risk triage and improving clinical confidence. Finally, when cytology is negative and methylation is positive (observed in 82 cases in this study, 74 of which were confirmed malignant), the high PPV of 90.2% for methylation in this context strongly suggests malignancy and warrants close clinical follow-up or repeat diagnostic confirmation (Figure 6).

This study establishes the first validation of the LungMe^®^ assay across a wide range of cancers causing both pleural and peritoneal effusions, moving beyond its primary focus on lung cancer. The high and consistent sensitivity observed in lung, breast, gastric, ovarian, and lymphomatous effusions underscores its broad applicability. When analyzed separately, *SHOX2* methylation alone exhibited high sensitivity for most tumor types (53.8–100%), while *RASSF1A* showed low overall sensitivity (7.3–38.8%) except in breast cancer (78.6%). The observed variability in methylation sensitivity across tumor types likely reflects several underlying biological factors, among which epigenetic heterogeneity plays a crucial role. For instance, both *SHOX2* and *RASSF1A* markers occupy different positions in tumorigenesis; the *SHOX2* gene’s promoter is generally unmethylated in mature somatic tissues and becomes hypermethylated as a comparatively early epigenetic event across many carcinomas [26], whereas *RASSF1A* hypermethylation varies more widely between lineages and has increasingly become an established independent biomarker linked to tumor invasiveness, metastasis, and poor prognosis [20]. This lineage dependence is directly evident in our cohort, where *RASSF1A* positively ranged from 78.6% among breast cancer effusions to 7.3% in gastric cancer effusions, a more than tenfold difference that parallels the tissue-specific frequencies of *RASSF1A* promoter methylation reported in primary tumors [16]. Notably, the complementary effect of the dual-marker combination was particularly evident for breast, lung, and hepatocellular carcinomas, the lineages in which *RASSF1A* methylation is most frequent and which tend to be more aggressive and metastatic, experience aberrant promoter hypermethylation, and are associated with malignant effusions [27]. On the other hand, sensitivity was lower for colorectal cancer (53.8%), which could be improved by incorporating additional markers such as *SEPTIN9*, a highly sensitive methylation biomarker for colorectal cancer [28], or *HOXA9* [29].

Variability in *SHOX2*/*RASSF1A* methylation detection across malignancies may also reflect tumor-specific factors, including unique cellular shedding patterns and serosal niches [30]. Especially since *SHOX2* promoter hypermethylation specifically tends to be an early and lineage-broad event, the reduced sensitivity observed in colorectal (53.8%) and ovarian (59.3%) effusions is unlikely to be attributed to epigenetic patterns alone, and may point instead to differences in tumor DNA dissemination into the serosal cavity. The efficacy of tumor cells shedding into pleural and/or peritoneal cavities is largely dictated by anatomical access and proximity of the tumor to a serosal surface, by the depth of serosal invasion, and by the migratory and metastatic potential of the tumor cells. Tumors contiguous with or directly invading the serosal cavity deliver tumor DNA to the pleural/peritoneal cavities more efficiently, which is consistent with our observation of lung and breast cancer, whose tumors are often adjacent to a serosal cavity with sensitivities of 80.1% and 100% respectively for LungMe^®^-positive effusions. Conversely, desmoplastic, mucin-rich peritoneal deposits characteristic of colorectal, ovarian, and gastric cancers are embedded in dense extracellular matrix that may restrict the release of intact tumor cells, limiting the detection of methylated tumor populations [30]. Pleural and peritoneal effusions are also not biologically similar niches, differing in mesothelial activation, stromal and extracellular matrix remodeling, and immune-cell infiltration [31]. Niche composition itself can modulate the apparent signal since ΔCt is normalized to β-actin from all nucleated cells, including abundant mesothelial and inflammatory cells that contribute unmethylated background DNA that can mask a methylated tumor population. This is a plausible contributor to the reduced sensitivity in ovarian carcinoma (73.1%), which is characterized by cellular- and inflammation-rich ascites. Methylation, nonetheless, appeared largely resilient to these differences across malignancies, with a sensitivity difference between pleural and peritoneal effusions of 8.7%, 10.8%, and only 3.6% for TCT, cell block, and LungMe^®^ *SHOX2*/*RASSF1A* methylation assay analyses respectively.

**Figure 7:**
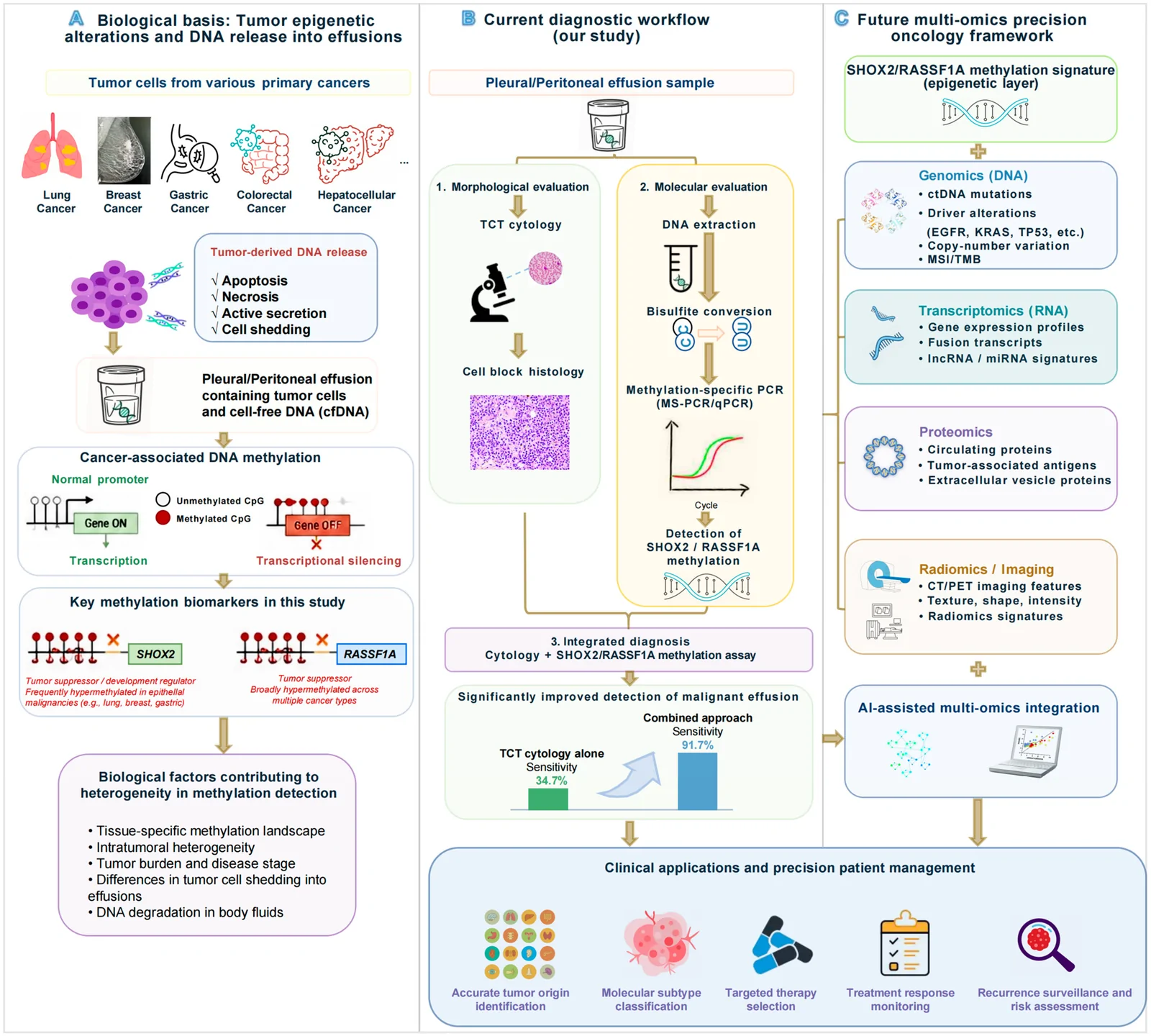
Proposed SHOX2/RASSF1A methylation-based multi-omics precision diagnostic framework for malignant pleural and peritoneal effusions.

Furthermore, our study used DNA extracted from the cellular pellet of effusion samples, which represents exfoliated cells from normal and neoplastic tissues. This approach yielded robust diagnostic performance. However, cell-free DNA (cfDNA) from the supernatant (originating from necrotic, fragmented, or secreted extracellular DNA from tumor cells, adjacent tissues, or metastatic lesions) may offer different, potentially complementary, diagnostic and prognostic information. In lung adenocarcinoma patients with malignant pleural effusions, cfDNA has demonstrated high clinical utility, with mutation detection sensitivity reaching 90.7%, surpassing even tissue biopsy (88%) [32]. Determining the optimal source of DNA for methylation analysis, whether from supernatant, cellular pellet, or a combination of both, will be critical for maximizing diagnostic accuracy, particularly for tumor staging and prognostic evaluation. Indeed, just as site-specific liquid biopsies (e.g., cerebrospinal fluid) are imperative for delineating the spatial heterogeneity and dynamic genomic evolution of distant metastases, the molecular interrogation of regional effusions provides a uniquely accurate, minimally invasive avenue for real-time cancer monitoring and personalized therapeutic intervention [33]. An important limitation of the present assay is that it relies on only two methylation markers, *SHOX2* and *RASSF1A*, which may not fully capture the substantial molecular heterogeneity of malignant pleural and peritoneal effusions across different tumor types. Accordingly, the current two-gene assay should be regarded as a clinically feasible molecular adjunct rather than a comprehensive biomarker panel. Expanding the number and types of biomarkers may further improve diagnostic performance. Beyond cell-free DNA, other liquid biopsy components, particularly small extracellular vesicles carrying tumor-derived DNA, RNA, and proteins, are increasingly recognized as complementary sources of cancer-specific molecular information. Future integration of DNA methylation markers with extracellular vesicle-based biomarkers and other multimodal liquid biopsy approaches may therefore improve the sensitivity, robustness, and clinical utility of malignant effusion diagnosis [34]. Beyond improving the sensitivity of malignant effusion diagnosis, the present findings may provide an epigenetic foundation for future multimodal precision-oncology strategies, as summarized in Figure 7.

This study has several limitations that should be acknowledged. First, this was a single-center study without external validation, and the predominance of lung cancer among pleural effusions may have inadvertently introduced spectrum bias, limiting generalizability to populations with different cancer distributions. Second, the ΔCt cutoffs were derived using ROC analysis on the same dataset used for performance analysis, which may have led to overfitting, although the utilized ΔCt cutoffs did align with previously reported values [22,23]. Third, complete blinding of cytopathologists to methylation results and of molecular analysts to clinical diagnoses was not explicitly implemented, which could have introduced interpretation bias. Fourth, sensitivity varied widely by tumor type, being lowest for colorectal (53.8%) and hepatocellular (71.0%) carcinomas, while subgroup sizes for rarer cancers were small (esophageal, *n* = 7; cholangiocarcinoma, *n* = 7), resulting in wide confidence intervals that warrant cautious interpretation of certain tumor-specific results (Table 3). Fifth, tumor cell percentage in sediment was not quantified, and the LungMe^®^ assay was not compared against broader methylation panels or NGS-based approaches. Finally, this study was not designed to assess cost effectiveness or evaluate the impact of methylation results on clinical decision-making.

Nevertheless, our findings open doors to many future directions, including the need for a multicenter validation study, which we are planning, that will confirm generalizability and the robustness of the ΔCt cutoffs in an independent cohort with a different case mix. The LungMe^®^ methylation assay may also carry value beyond diagnosis, with previous studies supporting the association of unmethylated *RASSF1A* with disease progression in non-small cell lung cancer treated with chemotherapy [32]. Notably, *RASSF1A* was unmethylated in approximately 60% of the lung cancer effusions in this cohort, a substantial subgroup in which the prognostic and predictive value of this readout could be tested. More broadly, since the LungMe^®^ assay requires low DNA input and returns results within two days, it could be incorporated into current multi-omic workups already applied to effusions, providing essential triage for identifying specimens with sufficient tumor DNA for downstream sequencing. Thus, prospective studies pairing effusion methylation with genetic profiling, transcriptomic characterization of the tumor microenvironment, and radiomic assessment of disease burden should determine whether such integration improves individual patient management.

## 5. Conclusions

Multi-marker methylation profiling represents a promising advance in the diagnostic evaluation of pleural and peritoneal effusions. Our study demonstrates that combined *SHOX2* and *RASSF1A* methylation testing effectively distinguishes benign from malignant effusions across diverse cancer types. When integrated with cytomorphological analysis, this approach substantially enhances tumor detection sensitivity, supporting a potentially more accurate and minimally invasive diagnostic paradigm. Yet, while this strategy holds broad clinical promise for early cancer detection, population screening, high-risk triage, and adjunctive diagnostics, the findings presented here are constrained by the single-center design, predominance of lung cancer pleural effusions, and small sample sizes for certain tumor subtypes, requiring further multicenter external validation and prospective clinical studies before routine clinical adoption.

## Figures and Tables

**Table 1:** Baseline characteristics of patients.

Characteristic		Pleural Effusions (N = 476)		Peritoneal Effusions (N = 220)		Pleural and Peritoneal Effusions (N = 696)	
		Malignant (%)	Benign (%)	Malignant (%)	Benign (%)	Malignant (%)	Benign (%)
		(*n* = 271)	(*n* = 205)	(*n* = 164)	(*n* = 56)	(*n* = 435)	(*n* = 261)
Gender	Male	156 (57.6)	142 (69.3)	78 (47.6)	31 (55.4)	234 (53.8)	173 (66.3)
	Female	115 (42.4)	63 (30.7)	86 (52.4)	25 (41.6)	201 (46.2)	88 (33.7)
Age	(year)	68.8 ± 11.7	65.1 ± 16.8	64.6 ± 12.5	64.1 ± 16.2	67.2 ± 12.1	64.8 ± 16.6
	<50	12 (4.4)	39 (19.0)	14 (8.5)	13 (23.2)	26 (6.0)	52 (19.9)
	50–59	52 (19.2)	29 (14.1)	39 (23.8)	8 (14.3)	91(20.9)	37 (14.2)
	60–69	61 (22.5)	47 (22.9)	49 (29.9)	13 (23.2)	110 (25.3)	60 (23.0)
	70–79	97 (35.8)	47 (22.9)	41 (25.0)	12 (21.4)	138 (31.7)	59 (22.6)
	≥80	49 (18.1)	43 (21.0)	21 (12.8)	10 (17.9)	70 (16.1)	53 (20.3)

**Table 2:** Diagnostic performance of cytology and methylation testing alone or in combination to diagnose benign vs. malignant pleural and peritoneal effusions.

Test Item	Testing Result	Overall Positive Rate	Malignant Effusion (Sensitivity)	Benign Effusion (Specificity)		
		(*n* = 696)	(*n* = 435)	(*n* = 261)	PPV ^a^	NPV ^b^
Cell paraffin block	Cancer cells (+)	293 (42.1%)	292 (67.1%)	1 (99.6%)	99.7%	64.5%
Cytological TCT	Cancer cells (+)	151 (21.7%)	151 (34.7%)	0 (100%)	100.0%	47.9%
LungMe^®^ methylation	Positive (+)	369 (53.0%)	344 (79.1%)	25 (90.4%)	93.2%	72.2%
	Strongly positive (++)	246 (35.3%)	246 (56.6%)	0 (100%)	100%	58.0%
Combinatory	Cell paraffin block + TCT + LungMe^®^ (+)	425 (61.1%)	399 (91.7%)	26 (90.0%)	93.9%	86.7%

^a^ PPV, Positive predictive value. ^b^ NPV, Negative predictive value.

**Table 3:** Diagnostic value of combined methylation assay LungMe^®^ for malignant pleural and peritoneal effusions caused by different tumor.

Tumor Type	Pleural Effusions (*n* = 476)			Peritoneal Effusions (*n* = 220)			Pleural and Peritoneal Effusions (*n* = 696)		
		LungMe^®^ (+)	Sensitivity		LungMe^®^ (+)	Sensitivity		LungMe^®^ (+)	Sensitivity
	*n*	*n*	(%)	*n*	*n*	(%)	*n*	*n*	(%)
Esophagus cancer	6	6	(100)	1	1	(100)	7	7	(100)
Breast cancer	9	9	(100)	5	5	(100)	14	14	(100)
Lung cancer	195	156	(80.0)	6	5	(83.3)	201	161	(80.1)
Gastric cancer	15	12	(80.0)	26	22	(84.6)	41	34	(82.9)
Lymphoma	13	11	(84.6)	4	3	(75.0)	17	14	(82.4)
Cholangiocarcinoma	1	1	(100)	6	5	(83.3)	7	6	(85.7)
Ovarian cancer	9	7	(77.8)	43	31	(72.1)	52	38	(73.1)
Liver cancer	5	3	(60.0)	26	19	(73.1)	31	22	(71.0)
Pancreatic cancer	4	2	(50.0)	14	12	(85.7)	18	14	(77.8)
Colorectal cancer	5	3	(60.0)	8	4	(50.0)	13	7	(53.8)
Other malignancies	9	8	(88.9)	25	19	(76.0)	34	27	(79.4)
Total	271	218	(80.4)	164	126	(76.8)	435	344	(79.1)

LungMe^®^ (+), positive LungMe^®^ methylation result.

## Data Availability

The datasets generated and analyzed during the current study are available from the corresponding author upon reasonable request. The data are not publicly available because they contain confidential clinical information and are subject to privacy protection and institutional ethical restrictions.

## Supplementary Materials

The following supporting information can be downloaded at: https://www.mdpi.com/article/10.3390/cancers18193176/s1, Table S1: Diagnostic Performance of LungMe^®^ in 272 Patients With Cytology Negative for Malignant Cells. Table S2: Diagnostic Performance of LungMe^®^ in 273 Patients With Equivocal Cytology.
