# Supplementary material for: A Dual-Marker DNA Methylation Assay Enables High-Sensitivity Detection of Malignant Effusions Across Cancer Types
Source: Cancers (Basel). 2026 Oct 1;18(19):3176. doi: 10.3390/cancers18193176 (PMC13664152; doi:10.3390/cancers18193176)
Supplement: Supplementary file 1 [file cancers-18-03176-s001.zip › cancers-4446833-supplementary.pdf]

**Supplementary Table S1. Diagnostic Performance of LungMe® in 272 Patients With Cytology Negative for Malignant Cells**

| LungMe® | Malignant | Benign | Total |
|---|---|---|---|
| Total | 97 | 175 | 272 |
| Positive | 74 (76.3%) | 8 (4.6%) | 82 |
| Negative | 23 (23.7%) | 167 (95.4%) | 190 |

**Supplementary Table S2. Diagnostic Performance of LungMe® in 273 Patients With Equivocal Cytology**

| LungMe® | Malignant | Benign | Total |
|---|---|---|---|
| Total | 187 | 86 | 273 |
| Positive | 147 (78.6%) | 17 (19.8%) | 164 |
| Negative | 40 (21.4%) | 69 (80.2%) | 109 |
